# *In Vitro* and *in Vivo* Evaluation of the Antioxidant and Prooxidant Activity of Phenolic Compounds Obtained from Grape (*Vitis vinifera)* Pomace

**DOI:** 10.3390/molecules191221154

**Published:** 2014-12-16

**Authors:** Milena Cotoras, Herman Vivanco, Ricardo Melo, María Aguirre, Evelyn Silva, Leonora Mendoza

**Affiliations:** 1 Laboratorio de Micología, Facultad de Química y Biología, Universidad de Santiago de Chile, Alameda 3363, Estación Central, Santiago 916000, Chile; E-Mails: herman.vivancov@usach.cl (H.V.); ricardo.melo@usach.cl (R.M.); evelyn.silva@usach.cl (E.S.); 2 Laboratorio de Electroquímica, Facultad de Química y Biología, Universidad de Santiago de Chile, Alameda 3363, Estación Central, Santiago 916000, Chile; E-Mail: maria.aguirre@usach.cl

**Keywords:** grape pomace, phenolic compounds, antioxidant and prooxidant activity

## Abstract

The antioxidant and/or prooxidant ability of extracts obtained from wine waste were analyzed using *in vitro* and *in vivo* assays. Cyclic voltammetry was used as the *in vitro* assay to determine the antioxidant and/or prooxidant properties and, the *in vivo* effect on mycelial growth of the fungus *Botrytis cinerea* was evaluated. In addition, the prooxidant activity was evaluated by intracellular oxidation of compound 2,7-dichlorodihydrofluorescein diacetate (DCFH-DA) in *B. cinerea*. The extracts used in this study were obtained from grape pomace of Cabernet Sauvignon, Carménère and Syrah varieties from the Misiones de Rengo Vineyard by simple extraction, using methanol/HCl 1% (v/v), ethanol 70% (v/v), or Soxhlet extraction. According to the results obtained, gallic acid was the most represented phenolic compound independent of grape variety and extraction method. In addition, vanillic acid; protocatechuic acid, syringic acid, quercetin and kaempferol were found in the extracts. From this study it was possible concluded that, depending of the method of extraction of the grape residues and the grape variety (Cabernet Sauvignon, Carménère and Syrah), the extracts showed antioxidant and/or prooxidant activity. However, no correlation can be established between the anodic oxidation potentials of the extracts and their effect on the fungus *B. cinerea*.

## 1. Introduction

Wine industry produces two types of wastes, wastewater and organic solid wastes generating a noticeable environmental impact [1]. The main organic wastes produced in modern wine industries include grape pomace (62%), lees (14%), stalk (12%) and dewatered sludge (12%). Currently, only a minimal amount of these residues are recycled [2].

Grape pomace consists mainly of skin residues, broken cells with pulp remains, stalks, and seeds [1]. Its composition varies depending on grape variety climate, culture localization and technology of vinification [3]. In addition, it has been described that grape pomace contains a great amount of anthocyanins, catechins, flavonoles, alcohols, stilbenes and phenolic compounds. These last compounds have pharmaceutical and agronomical applications since antioxidant and free radical scavenging, anticancer, antifungal and antibacterial activities have been reported. Among these biological properties, the best known and most widely studied is the antioxidant effect, which has been the focus of a large amount of analysis, mostly of clinical and nutritional nature [4].

In particular, it has been described that citrus peel [5], apple and grape pomace [6,7] contain several phenolic compounds. Among them catechins (catechin, epicatechin), flavonols (quercetin, kaempferol, myricetin), benzoic acids (gallic, protocatechuic, 4-hydroxybenzoic, syringic, gentisic) and cinnamic acids (*p*-coumaric) are representative [8]. Recently, Mendoza *et al.* [9] compared different extraction techniques to obtain grape pomace extracts with antifungal activity. These authors reported that quercetin was a phenolic compound most representative from a mixture of Chilean grape varieties (Cabernet Sauvignon, Carmènere and Syrah). However, kaempferol, vanillic and syringic acid were also identified. From this study, it was possible conclude that the grape pomace can be considered as a good low cost source to obtain extracts with antifungal activity against the phytophatogenic fungus *Botrytis cinerea*. This fungus, the causal agent of gray mould, is a broad host pathogen that derives its sustenance from dead or necrotic plants. In particular, the infections produced by *Botrytis* result in great economic losses, not only during growth but also during storage and transport. In particular, grapes are one of the commodities most affected by *Botrytis* infection [10].

Characterizations of phenolic compounds have pointed them out as powerful *in vitro* antioxidants, even more potent than Vitamins C and E and the carotenoids [11,12]. However, it was also found that phenolic antioxidants behave like prooxidants under the conditions that favor their autoxidation, for example, at high pH with high concentrations of transition metal ions and oxygen molecules present. Small phenolics that are easily oxidized, such as quercetin, gallic acid, possess prooxidant activity; while high molecular weight phenolics, such as condensed and hydrolysable tannins, have little or no prooxidant activity [13]. Supporting this observation, it has been described that quercetin and caffeic acid take part in redox reactions in which they can act as either antioxidants (electron donors) or prooxidants (electron acceptors), depending on their environment. In addition, Fukumoto and Mazza noted dual antioxidant and prooxidant activities for a variety of plant-derived polyphenols, including gallic acid, protocatechuic acid, syringic acid, vanillic acid, ellagic acid, caffeic acid, coumaric acid, chlorogenic acid, ferulic acid, myricetin, quercetin, rutin, kaempferol, (+)-catechin, (‒)-epicatechin, delphinidin, and malvidin [14]. Therefore, the prooxidant activity of phenolic compounds would explain the antifungal effect previously reported [9]. In *Candida* spp., it has been shown that the phenolic compound curcumin increased the reactive oxygen species level and induced early apoptosis [15]. In *Rhyzopus spp* and in *Aspergillus* the fluidity of the membrane was disrupted by interaction of phenolic compounds with ergosterol [16,17]. In *B. cinerea* the 5,7-dihydroxy-3,8-dimethoxyflavone partially affected conidial germination, reduced oxygen consumption and, affected the plasma membrane integrity [18].

Hence, this work aimed to the achieve the following: (i) analyze phenolic compounds obtained from different pomace grape varieties by different extraction methods; (ii) determine the *in vitro* antioxidant/prooxidant activity from each extract by voltammetry cyclic and (iii) evaluate the *in vivo* prooxidant activity using *B. cinerea* as model organism.

## 2. Results and Discussion

### 2.1. Analysis of Antioxidant and Prooxidant Activity and Phenolic Compound Composition from Different Pomace Grape Varieties

In this work, whole or ground grape pomace samples from the varieties Cabernet Sauvignon, Carménère and Syrah and two-extraction methods (solid-liquid extraction and Soxhlet) were tested to obtain polyphenol-enriched extracts (Table 1). Later, each extract was submitted to liquid-liquid extraction using different solvent as a hexane, methanol, chloroform and ethyl acetate. The higher extraction yield, in all grape varieties (Cabernet Sauvignon, Carménère and Syrah) was obtained by means of simple extraction method using methanol/HCl 1% (v/v). These results are concordant with the described by Castañeda-Ovando *et al.* [19]. According to the type or sample of pomace grape, the highest yield (30%) was obtained from ground pomace Cabernet Sauvignon by simple extraction instead of Soxhlet extraction which only produced a maximum of 12% yield in all strains (data not shown). On the other hand, the total phenol concentration in the different fractions varied among 300 and 1000 mg/mL. There was no correlation between the extraction method or the grape pomace variety and the total phenol concentration (data not shown). In addition, phenolic compounds in the different fractions were identified by HPLC. The results are also shown in Table 1.

It has been widely reported that the extraction method can affect the phenolic profile of the resulting extracts [4]. In addition, it has been also described that extracts obtained by liquid extraction from whole substrates showed lower yields than from ground substrates [9]. Based on the previous results, the presence of different phenolic compounds in different fractions was not a surprise. As might be expected, several phenolic compounds were identified. Among them, gallic acid was the most represented. This phenolic acid was present independent of grape variety and extraction method. It has been reported that gallic acid is one of the major phenolic compounds founded in grape seeds and skins together with catechin and epicatechin [20]. Another highly represented phenolic compound was vanillic acid. However, this compound was lacking in the grape varieties treated with ethanol–system 2. Interestingly, protocatechuic acid was also highly represented in the different grape varieties independent of extraction method but, like vanillic acid, it was not possible to identify this compound on the fraction extracted with ethanol-system 2. It has been described that protocatechuic acid has an antioxidant effect due to its capacity to chelate metal ions and scavenge free radicals [21]. On the other hand, 4-hydroxyphenylacetic acid was identified only in the fraction extracted with ethanol system 2. Other compounds identified were syringic acid, quercetin and kaempferol, evenly distributed across the extraction methods.

**Table 1:** Pomace grape variety and extraction; oxidation potentials, chemistry composition from grape pomace extracts using different extraction methods. Epa corresponds to anodic oxidation potential and script a and b represents different oxidation peaks.

Extraction Method	Grape Variety	Fraction	Sample	Epa (V) ^a^	Epa (V) ^b^	Phenolic Composition
**Methanol/HCl**	Cabernet Sauvignon	Ethyl acetate	Ground	0.4	0.75	vanillic acid, syringic acid, gallic acid, kaempferol, quercetin, protocatechuic
	Carménère		Whole	0.4	0.8	vanillic acid, syringic acid, gallic acid, elagic acid, quercetin, 4-hydroxi-3,5-dimethoxibenzaldehyde
	Syrah		Ground	0.35	0.7	gallic acid, p-coumaric, elagic acid, quercetin, kaempferol
			Whole	0.4	0.8	gallic acid, protocatechuic, vanillic acid, syringic acid, quercetin, kaempferol
**Ethanol (System 1)**	Cabernet Sauvignon	Hexane	Whole	-	0.6	-
			Ground	0.35	-	gallic acid, vanillic acid, syringic acid, (−) epicatechin, quercetin, kaempferol
		Chloroform	Whole	-	0.7	gallic acid, protocatechuic acid, vanillic acid, syringic acid, elagic acid, quercetin, kaempferol
		Ethyl acetate	Ground	-	0.6	gallic acid, (−) epicatechin, quercetin
	Carmènére	Chloroform	Ground	0.3	-	vanillic acid, syringic acid, quercetin, kaempferol
			Whole	0.41	-	vanillic acid, syringic acid, quercetin, kaempferol
		Ethyl acetate		0.4	-	gallic acid, protocatechuic acid, quercetin, (−) epicatechin
	Syrah	Chloroform	Ground	0.38	-	gallic acid, vanillic acid, syringic acid, quercetin, kaempferol
			Whole	0.34	-	vanillic acid, syringic acid, quercetin, kaempferol
		Ethyl acetate	Ground	0.3	-	gallic acid, vanillic acid, syringic acid, quercetin, (−) Epicatechin
			Whole	0.35	0.8	gallic, protocatechuic acid, vanillic acid, p-coumárico, Elagic acid
**Ethanol (System 2)**	Cabernet Sauvignon	Chloroform	Ground	-	0.58	syringic acid, quercetin,kaempferol, 4-hydroxiphenyl acetic acid
			Whole	-	0.57	4-hydroxiphenil acetic acid, syringic acid, quercetin, kaempferol
		Ethyl acetate	Ground	0.31	-	gallic acid, quercetin, (−) Epicatechin
	Carménère	Chloroform	Ground	0.29	-	gallic acid, Catequin, quercetin, (−) Epicatechin
			Whole	-	0.55	gallic acid, Catequin, quercetin, (−) Epicatechin
			Ground	0.34	-	-
	Syrah	Hexane	Whole	-	0.6	quercetin
		Chloroform	Ground	0.32	-	4-hydroxiphenil acetic acid, syringic acid, quercetin, kaempferol
		Ethyl acetate	Ground	0.33	-	gallic acid, vanillic acid, quercetin, (−) Epicatechin

Currently several authors have described that phenolic compounds extracted from plants materials, have antioxidative properties in various model systems and in several foods, where they are finding increasing use [22,23,24]. However, it has been also described that these compounds can exhibit prooxidant and cytotoxic properties under certain conditions such as the presence of metals, pH, structural characteristics and concentration [25]. On the other hand, it has been shown that mixtures of different phenolic compounds can produce synergistic or antagonistic prooxidant effects [26]. Due to the fact a mixture of phenolic compounds is found in the grape pomace extracts, in this work, antioxidant and prooxidant activities of extracts obtained from *V. vinifera* residues were determined using different approaches.

**Figure 1:**
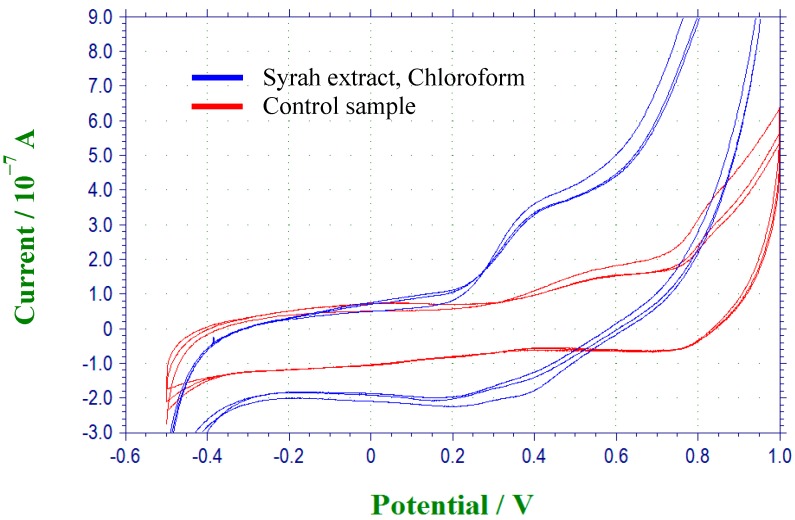
Voltammogram of chloroform extract from Syrah (350 ppm) dissolved in DMF/0.1 M TBAP at 10 mV/s.

In the first one, the antioxidant and prooxidant activity was determined *in vitro* by a cyclic voltammetry assay using the criteria described by Simić *et al.* These authors established that compounds with low oxidation potentials (anodic oxidation potential (Epa) lower than 0.45) showed antioxidant activity, whereas compounds with high Epa values (>0.45) acted as prooxidants [27]. Figure 1 shows an example of a voltammogram corresponding to the chloroform extract from Syrah variety obtained by solid-liquid extraction using 70% ethanol (v/v) (system 1), where an anodic oxidation peak is observed (Epa) at 0.38 V.

Based on this technique, the antioxidant or prooxidant ability of grape pomace extracts were obtained and some values are listed in the Table 1, where Epa corresponds to the anodic oxidation potential and the subscripts (a) and (b) represent different oxidation peaks. It is necessary to comment that only those fractions that showed cyclic voltammetry signals are represented. According to the results shown in Table 1, it is possible to observe that in the methanol extraction from grape varieties, only the ethyl acetate fraction showed signals by cyclic voltammetry. These results could be explained because this solvent (ethyl acetate) presents higher polarity than the other solvents used, allowing a better extraction of phenolic compounds that can be oxidized and reduced in that range. Therefore, these extracts would present both antioxidant and prooxidant activities. An explanation could be that, in general, extracts that exhibited low oxidation potential values contained easily oxidized phenolic compounds such us quercetin, kaempferol, elagic acid, and protocatechuic according to describe by Simić *et al.* These authors investigated the electrochemical oxidation of a number natural phenolics (salicylic acid, *m*-hydroxybenzoic acid, *p*-hydroxybenzoic acid, protocatechuic acid, *o*-coumaric acid, *m*-coumaric acid, *p*-coumaric acid, caffeic acid, quercetin and rutin) suggesting that multiple OH substitution and conjugation are important determinants of the free radical scavenging activity and electrochemical behavior. As we mentioned previously, compounds with low oxidation potentials showed antioxidant activity, whereas compounds with high Epa values act as prooxidants. This characteristic could explain an antagonistic interaction with phenolic compounds that present higher oxidation potential values (vanillin and syringic acid) present in some extracts simultaneously displaying antioxidant and prooxidant activity. In our case, the phenolic compounds present in the fraction treated with methanol/HCl extraction are similar those mentioned by Simić *et al.* [27].

On the other hand, the ethanol extractions (system 1 and system 2) showed differences in antioxidant or prooxidant activity for the different grape varieties as determined by cyclic voltammetry. System 1 presented a greater presence of fractions with only antioxidant ability than system 2 where the presence of antioxidant and prooxidant ability appears to be balanced. If grape varieties are compared, it is possible to observe that the Cabernet Sauvignon variety presented mainly a prooxidant activity, except when the extracts were obtained with chloroform where oxidant ability is present (ground sample). The activity exhibited by Cabernet Sauvignon variety could be attributed to the presence of other compounds with properties to oxidize in the range measured by cyclic voltammetry despite the fact phenolic compounds were not detected in this fraction. Besides that factors that could clarity the oxidant activity in the chloroform fraction are the presence of vanillic and syringic acids identified in these fractions. Supporting this observation, it has been described that the methylation of the 3-hydroxyl group in protocatechuic acid to form vanillic acid caused a significant reduction of the radical scavenging capacity and the introduction of another methoxy group in the 5-position to form syringic acid slightly decreased the prooxidant activity (Simić *et al.* [27]). Regarding the Carménère extracts, they presented antioxidant activity independent of the solvent used and prooxidant activity was founded only in a fraction treated with chloroform. With respect to the Syrah variety, all fractions showed antioxidant activity, except the hexane fraction in the system 2 that presented a prooxidant activity. These findings could be explained based on the fact that this fraction contains phenolic compounds such as catechin, quercetin, kaempferol and gallic acid for which higher levels of antioxidant activity have been reported compared with those with a single hydroxyl group [28].

### 2.2. Evaluation of in Vivo Prooxidant Ability of the Extracts

It has been widely described that phenolic compounds originating from various plant sources are important anti-inflammatory, anti-histamine, antiviral, antibacterial and even antifungal agents due to their antioxidant or prooxidant capacity [28,29]. In the case of phenolic acids, the structure of these compounds is crucial because the antioxidant activity depends on the number and position of hydroxyl groups relative to the carboxyl functional group [12]. In this work, the *in vivo* prooxidant ability of the extracts was analyzed using the phytopathogenic fungus *B. cinerea* as a study model. For this, a series of extracts with antioxidant and/or prooxidant activity ability determined by cyclic voltammetry assay were selected to evaluate the *B. cinerea* growth under their presence. The results are presented in the Table 2.

**Table 2:** Effect of extracts of selected Carménère, Cabernet and Syrah varieties on mycelial growth of *Botrytis cinerea*. Epa corresponds to anodic oxidation potential and script a and b represents different oxidation peaks.

Extraction Solvent	Grape Variety	Fraction	Sample	Antioxidant Epa (V) ^a^	Prooxidant Epa (V) ^b^	ED_50_ ± SD (μg/mL)
Methanol/HCl	Carménère Syrah	Ethyl acetate	Whole	0.4 0.4	0.8 0.8	52.04 ± 3.32 50.06 ± 3.34
Ethanol 70%(System 1)	Cabernet Sauvignon Syrah	Chloroform	Ground Whole	0.35 0.38	- -	49.34 ± 6.24 50.76 ± 2.58
	Syrah	Ethyl acetate	Whole	0.35	0.8	44.59 ± 7.75
Ethanol 70%(System 2)	Cabernet Sauvignon	Ethyl acetate	Ground	0.31	-	41.97 ± 5.23
	Carménère	Chloroform	Whole	-	0.55	22.81 ± 3.28
	Syrah	Ethyl acetate	Ground	0.33	-	72.64 ± 7.27

It is possible to observe that all the fractions inhibited the hyphal growth of *B. cinerea* expressed in relation to IC_50_ (concentration that reduced mycelial growth by 50%). The higher antifungal activity was obtained from chloroform extract from Carménère variety extracted with 70% ethanol (system 2). This extract showed only an oxidation peak in the voltagramm corresponding to prooxidant activity and phenolic compounds as a gallic acid, catequin, quercetin and (−) epiquercetin were identified (data not shown). On the other hand, a lower effect was observed with the same pomace grape variety and extraction method but using ethyl acetate as a solvent. It should be noted that this fraction has a phenolic composition similar to that of the fraction with higher antifungal activity but additionally it contained vanillic acid. It is tempting to suggest that the inhibition depends on the presence of antioxidant polyphenols such as catequin, epicatequin and quercetin, which would enhance the antifungal effects. Supporting this observation, it has been described that catequin, a polyphenol very abundant in tea plants, is a strong radical scavenger and metal chelator in model chemical systems, and these effects correlate with the presence of the dihydroxy and trihydroxy groups [30]. In addition, an increasing number of studies have also demonstrated these antioxidative effects *in vivo* [28,29]. In contrast, the presence of vanillic acid, a phenolic compound with a high oxidation potential, unlike catequin or gallic acid [27,31] would produce less growth inhibition effect on *B. cinerea.*

**Figure 2:**
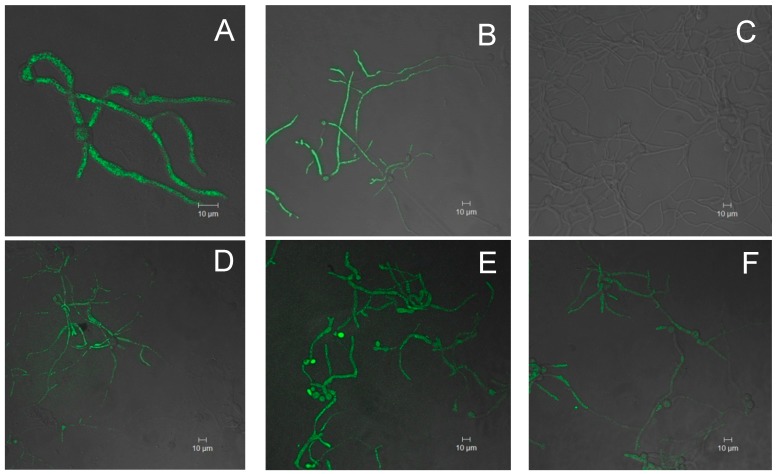
Detection of intracellular ROS by DCFH-DA in *B. cinerea* hyphae. (**A**,**B**) corresponds to positive controls, farnesol (100 mM) and H_2_O_2_ (300 mM) respectively; (**C**) negative control (methanol); (**D**) Carménère variety, ethyl acetate fraction (40 ppm) obtained by methanol/HCl 1% (v/v) extraction method; (**E**,**F**) chloroform fraction (40 ppm), strain and ethyl acetate (40 ppm) fraction from, Carménère and Syrah varieties respectively obtained by using ethanol 70% (v/v) (system 2). All the samples were incubated for 6 h.

To elucidate if the inhibition of *B. cinerea* mycelium growth in the presence of the extracts is due to the prooxidant activity of these components *in vivo* intracellular oxidation assays were performed. To analyze the intracellular oxidation, the DCFH-DA probe was used. This probe is cell permeable and is hydrolyzed by cellular esterases to the DCFH carboxylate anion, which is oxidized by reactive species to a fluorescent product, dichlorofluorescein (DCF) [32]. Fungal mycelia were incubated in presence of 40 ppm of the extracts for 6 h and the oxygen reactive species (ROS) registered. As a positive control farnesol was used. Farnesol is an isoprenoid alcohol that has been described to increase ROS levels, inhibit growth and induce cell death in yeast and fungi, including *B. cinerea*, *Aspergillus nidulans*, *Candida albicans* and *Saccharomyces cerevisiae* [33,34]. The results are presented in the Figure 2.

According to the results obtained from intracellular oxidation using the probe 2',7'-dichloro-dihydrofluorescein diacetate (DCFH-DA) it was possible to observe that all tested extracts presented an accumulation of reactive oxygen species (ROS) at 40 ppm demonstrating an oxidative stress, similar to that observed in the presence of farnesol (positive control). These results suggest that these extracts at the concentration tested, exerted a prooxidant effect on *B. cinerea* what would cause a toxic effect, inhibiting mycelial growth.

Therefore, based on the results obtained in this work, it is not possible to correlate antioxidant and/or prooxidant activity determined by cyclic voltammetry with *B. cinerea* mycelial growth inhibition. Probably, because multiple variables are involved in the *in vivo* assay as different mechanisms of action producing cell death by membrane damage, stress, *etc.* and the antioxidant cellular mechanisms which are not considered by the *in vitro* tests.

## 3. Experimental Section

### 3.1. Grape Pomaces

Pomaces were obtained after grape fermentation from *V. vinifera* varieties (Cabernet Sauvignon, Carménère and Syrah) from the 2009 harvest season from Misiones de Rengo Vineyard (Rengo, Chile). Pomaces were maintained at −20 °C until they were used.

### 3.2. General Extraction Process

Extracts were prepared from whole and ground grape pomace and the phenolic compounds were extracted by three different systems:

(A) *Methanol extraction*: Whole and ground grape pomace (400 g) were extracted with methanol/HCl (1% v/v, 1.5 L) for 4 h with constant agitation at 4 °C. Then extracts were concentrated in a rotary evaporator and distilled water (20 mL) was added and volume was decreased in a rotary evaporator. Water addition was repeated three times. Finally, aqueous suspension or crude extract was subjected to sequential liquid–liquid extraction with hexane, chloroform and finally ethyl acetate;

(B) *Ethanol extraction*: (i) System 1: Whole and ground grape pomace (400 g) were extracted with ethanol (70% v/v, 1.5 L) for 4 h with constant agitation at 4 °C. Extracts were concentrated in a rotary evaporator to afford crude extracts which were subjected to sequential liquid-liquid extraction with hexane, chloroform and finally ethyl acetate; (ii) System 2: whole and ground grape pomace (400 g) were extracted with ethanol (70% v/v, 1.5 L) for 4 h with constant agitation at 4 °C. Extracts were concentrated in a rotary evaporator and distilled water (20 mL) was added and then the volume was reduced on a rotary evaporator. Water addition was repeated three times. Finally, aqueous suspension or crude extract was subjected to sequential liquid–liquid extraction with hexane, chloroform and finally ethyl acetate;

(C) *Soxhlet*: whole grape pomace (10 g) was submitted to a Soxhlet extraction using methanol in a solid-liquid ratio of 1:20 at 40 °C for 12 h. Methanol was partially removed under reduced pressure. This methanol extract was subjected to liquid-liquid extraction with hexane, giving a methanolic and a hexane phase.

### 3.3. Determination of Total Phenol Content

The amount of total phenol, in different extracts, was determined by Folin-Ciocalteu’s reagent method. Total phenols content were expressed as gallic acid equivalent (milligrams per gram of extracted compounds) [35].

### 3.4. Analysis of Phenolic Compounds in Different Extractions

The HPLC analysis was performed as described by Mendoza *et al.* [36]. A Waters 600 HPLC chromatograph (Waters, Milford, MA, USA) equipped with a Waters 2990 diode array detector, and a Symmetry C-18 (5 μm, 3.9 mm × 150 mm) column (Waters) was used. The solvent system consisted of 1% aqueous formic acid (A) and 1% formic acid in acetonitrile (B). The initial composition of the mobile phase was 95% A and 5% B. With linear gradients the composition changed to 75% A and 25% B within 45 min, and 50% A and 50% B within 60 min. The flow rate was 0.8 mL/min. Identification of phenolic compounds was done by comparing their retention times and UV-Vis spectra with standards. Gallic acid, protocatechuic acid, vanillic acid, sinapic acid, syringic acid, ellagic acid, (+)-catechin, (−)-epicatechin, epigallocatechin, caffeic acid, vanillin, *p*-coumaric acid, 4-hydroxy-3,5-dimethoxybenzaldehyde, 4-hydroxyphenylacetic acid, transresveratrol, quercetin, kaempferol and myricetin were used as standards.

### 3.5. Determination in Vitro of Antioxidant or Prooxidant Activity of Grape Pomace Extracts by Cyclic Voltammetry

The antioxidant and prooxidant effects of extracts was determined using cyclic voltammetry as described by Simić *et al.* [27]. Electrochemical experiments were performed in a three-compartment glass cell, with a glassy carbon (A = 0.071 cm^2^) working electrode. Saturated Ag/AgCl/KCl was used as reference electrode with respect to which all the potentials are quoted, and a Pt coil (A = 10 cm^2^) was used as the counter electrode. The glassy carbon electrode was polished with 0.25 μm alumina and ultrasonicated for 5 min before each experiment. Oxidant and prooxidant behavior was determined by potentiodynamically cycling the electrode between −0.2 V and 2 V at a scan rate of 0.01 V/s in dichloromethane containing 350 μg/mL of the extracts in the presence of 0.1 M tetrabutylammonium perchlorate. The solution was purged with nitrogen (ultra-pure grade) during each measurement. All the experiments were carried out at room temperature in an inert atmosphere. Electrochemical measurements were performed using a CHI604C Bipotentiostat (CH Instruments, Austin, TX, USA) along with CH Instruments software.

### 3.6. Determination of in Vivo Prooxidant Activity of Grape Pomace Extracts

To determine the *in vivo* prooxidant activity of grape pomace extracts, the effect of the extracts on mycelial growth and on the production of oxidative stress in *B. cinerea* was evaluated.

#### 3.6.1. Fungal Isolate and Culture Conditions

In this study *B. cinerea* strain G29 was used. This strain was originally isolated from naturally infected grapes (*V. vinifera*) [37] and was maintained on malt-yeast extract agar slants (2% (*w*/*v*) malt extract, 0.2% (*w*/*v*) yeast extract and 1.5% (*w*/*v*) agar) at 4 °C. The fungus was grown in the dark on malt-yeast extract agar medium or soft agar medium (2% (*w*/*v*) malt extract, 0.2% (*w*/*v*) yeast extract and 0.6% (*w*/*v*) agar).

#### 3.6.2. Determination *in Vivo* Prooxidant Activity Using *B. cinerea* as Model Organism

The antifungal activity on mycelial growth of *B. cinerea* of different extracts and phenolic compounds was assessed using a protocol described by Mendoza *et al.* [9]. All the experiments were performed at least in triplicate and with adequate controls (solvent and positive controls). Additionally, a commercial fungicide was used as positive control. Antifungal effect results were expressed as IC_50_ (concentration that reduced mycelial growth by 50%) determined by regressing the inhibition of radial growth values (percent control) at different extract or compound concentration after 48 h of incubation.

To demonstrate *in vivo* prooxidant activity of some grape pomace extracts the probe 2,7-dichloro-dihydrofluorescein diacetate (DCFH-DA) in protoplasts of *B. cinerea* treated with 40 ppm of extracts for 6 h was used according to the manufacturer’s instructions. The protoplasts were obtained according the protocols described by Cotoras *et al.* [33]. After 15 min of incubation with the probe, the protoplasts attached to the coverslips were washed three times with minimal medium and mounted on slides according to describe by Cotoras *et al.* [33]. For mounting the samples DABCO (1,4-diazabicyclo[2.2.2]octane, 10 µL) was used. Finally, the protoplasts of *B. cinerea* were visualized under a confocal microscope (LSM 510, Carl Zeiss, Göttingen, Germany) at an excitation wavelength of 488 nm and an emission wavelength of 540 nm. Hyphae without exposure to oxidative stress were used as negative control; as for the positive control hyphae exposed to hydrogen peroxide (300 mM) or farnesol (100 mM) according Joo and Jetten [34] was used. Each experiment was performed in duplicate.

## 4. Conclusions

Depending on the method of extraction of the grape residues and the grape variety (Cabernet Sauvignon, Carménère and Syrah), the extracts show antioxidant and/or prooxidant activity. However, no correlation can be established between the anodic oxidation potentials of the extracts and their effect on the fungus *B. cinerea*.
